# The impact of CD4^+^CD28^null^ T lymphocytes on atrial fibrillation: a potential pathophysiological pathway

**DOI:** 10.1007/s00011-021-01502-w

**Published:** 2021-09-18

**Authors:** Andreas Hammer, Alexander Niessner, Patrick Sulzgruber

**Affiliations:** Division of Cardiology, Department of Internal Medicine II, Medical University of Vienna, Waehringer Guertel 18-20, 1090 Vienna, Austria

**Keywords:** Atrial fibrillation, CD4^+^CD28^null^, T lymphocytes

## Abstract

**Introduction:**

Atrial fibrillation (AF) represents the most common cardiac arrhythmia in daily clinical practice and substantially impacts affected patients by elevation of both morbidity and mortality. Previous investigations proved that inflammatory processes are closely linked to this multifactorial pathogenesis—especially autoreactive CD4^+^CD28^null^ T cells received in-depth attention.

**Purpose:**

Consequently, a potential pathophysiological pathway of the impact of CD4^+^CD28null T lymphocytes on the development and progression AF can be outlined.

**Conclusion:**

Considering the available data in the literature, it needs to be assumed that CD4^+^CD28^null^ T lymphocytes are mainly involved in the development of AF and disease progression. Of utmost importance, it can be considered as the result of a T-cell-mediated auto-immune reaction among myocardial tissue. However, mechanisms which recruit CD4^+^CD28^null^ cells in cardiac tissue remain unclear and need further investigation.

Chronic systemic inflammation seems to be a major pathophysiological pathway in the development and progression of atrial fibrillation (AF) [1]. Several investigations revealed that elevated levels of pro-inflammatory cytokines are closely associated with the occurrence of AF in the general population, and it is well known that a more pronounced inflammatory activity represents a strong risk factor of thromboembolic events in patients with AF independent of traditional clinical risk factors. Considering the well investigated association of inflammation and AF, T lymphocytes—especially *CD4*^+^*CD28*^+^ T lymphocytes—were recently named as promoters of interest in terms of the development and progression of AF.

The CD28 surface protein on CD4^+^CD28^+^ T lymphocytes represents a cell membrane receptor which is required for transduction of co-stimulatory signals via CD80 or CD86 and is therefore pivotal for the induction and maintenance of cellular immune response as well proliferation via both the expression of IL-2 receptors and release of IL-2. In case of absence of the signals delivered by co-stimulatory molecules the T cell remains in functional unresponsiveness [2]. However, during long-term chronic inflammation, and therefore based on repeated rounds of T cell activation, the CD28 surface protein becomes down regulated on several CD4^+^CD28^+^ T cells [3]. Mechanisms underlying this loss are still not known. This now called CD4^+^CD28^null^ T cell subset undergoes oligoclonal expansion. Moreover, this cell line is completely resistant to induction of apoptosis and their immune response cannot be suppressed by regulatory T cells. In this regard the T cell subset of CD4^+^CD28^null^ T cell accumulates over time [4].

While CD4^+^CD28^null^ T cells now differ from their conventional CD28^+^ counterparts in their phenotype, also the function of this T cell subset undergoes substantial changes. The CD28^null^ subset represents now a terminally differentiated T cell line and shows major pro-inflammatory potential characterized by the production of IFN-γ, IL-2 and TNF-α [5]. Moreover, they developed a cytotoxic potential and even showed aggressive behavior against endothelial tissue in vitro, which is predominantly mediated by perforin and granzymes [6].

Recent investigation revealed that CD4^+^CD28^null^ T cells were strongly and independently associated with the development of AF after cardiac surgery [7]. Moreover, CD4^+^CD28^null^ cells were found to be a strong and independent predictor of both all-cause mortality and cardiovascular mortality in patients presenting with chronic heart failure and AF, but were not predictive in patients free of AF [8]. Therefore, recently published data indicated a potential auto-immune impact of these highly cytotoxic T cell subset in the pathogenesis of AF. Besides their association with AF, CD4^+^CD28^null^ T cells have been recognized in several other inflammatory related diseases such as, rheumatoid arthritis, systemic lupus erythematosus, atherosclerosis, persistent viral infections (i.e. HIV, CMV), or even chronic kidney disease [9].

A potential pathophysiological pathway of the impact of CD4^+^CD28^null^ T lymphocytes and AF can be outlined as follows (see Fig. 1):

**Fig. 1:**
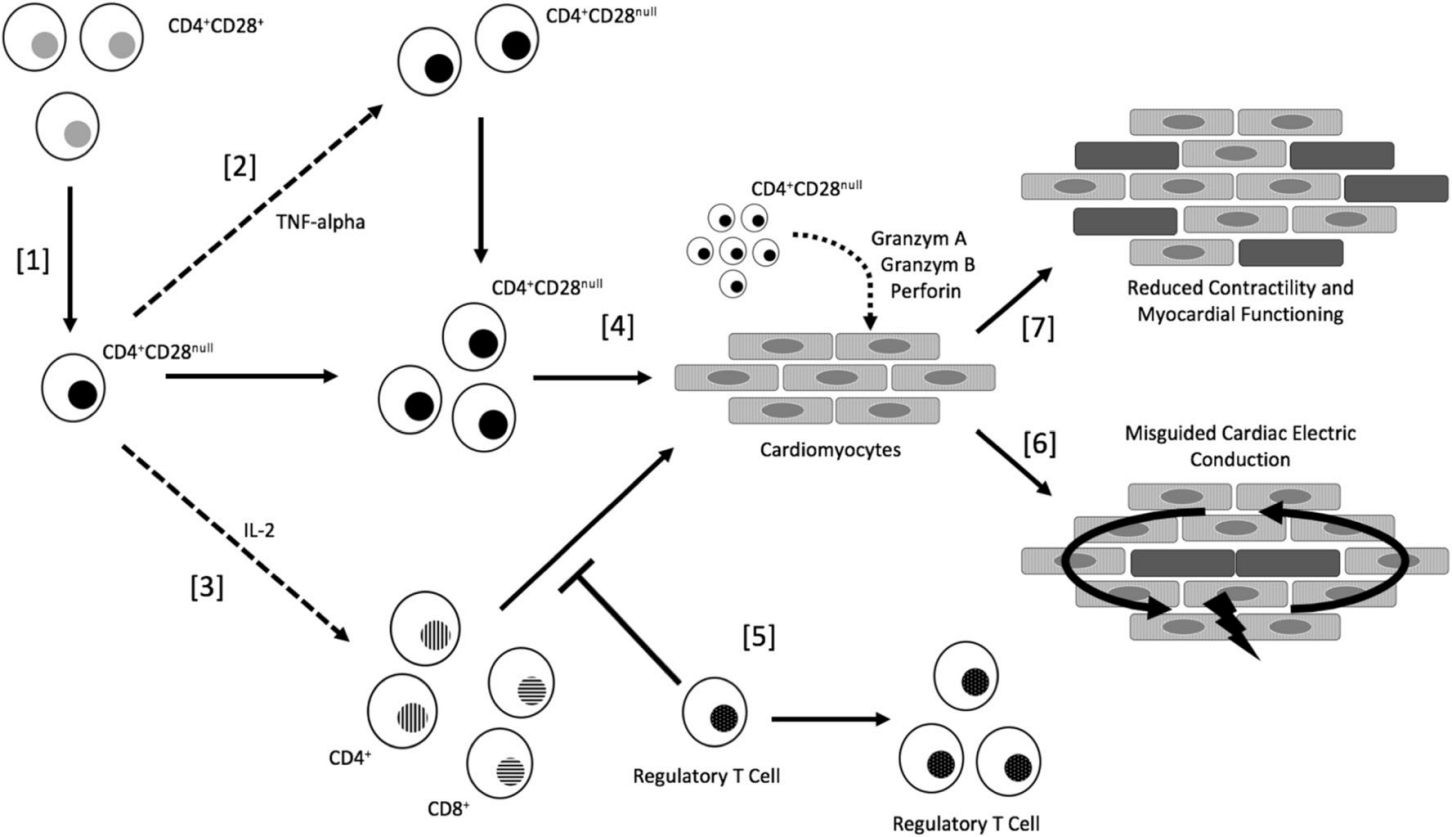
Hypothesized mechanism of the impact of CD4^+^CD28^null^ T lymphocytes on the development of atrial fibrillation and disease progression

[1]: Based on long-term cardiac inflammation the CD28 surface protein of CD4^+^CD28^+^ T lymphocytes gets lost. Possible triggers for cardiac inflammation such as hypertension, diabetes mellitus type 2, chronic heart failure, cardiac valve dysfunction, coronary artery disease or acute myocardial infarction—which activate cellular immunity resulting in cardiac remodeling and structural changes among myocardial tissue. The so-called CD4^+^CD28^null^ T cell subset now differs from their conventional CD28^+^ counterparts both in their phenotype and behavioral characteristics and undergoes oligoclonal expansion and increases its fraction during the course of inflammation and duration of disease [4, 10]. In this context, an elevated fraction of CD28^null^ cells within CD4^+^ cells could represent a strong surrogate parameter for both timely course of disease and severity of disease. (see Fig. 1—Sect. [1]).

[2]: The CD28^null^ subset represents now a terminally differentiated T cell line. It has pro-inflammatory potential via the release of TNF-α [5]. The pro-inflammatory molecule TNF-α is crucially involved in the expression and oligoclonal expansion of CD4^+^CD28^null^ cells, as well further recruitment and activation of this highly cytotoxic T cell subset. The expression of TNF-α is a key-function in the progression of CD4^+^CD28^null^ cell mediated inflammation. (see Fig. 1—Sect. [2]).

[3]: In parallel to the release of TNF-α, IL-2 is expressed by CD28^null^ cells [5]. The expression of IL-2 results in further T helper cell (CD4^+^) as well cytotoxic T cell (CD8^+^) and NK cell activation and recruitment at the site of inflammation. It therefore results in an aggravated state of inflammatory response among affected myocardial tissue. (see Fig. 1—Sect. [3]):

[4]: Based on the loss of the CD28 surface protein the CD4^+^CD28^null^ cells develop a cytotoxic potential and even showed aggressive behavior against endothelial tissue, which is predominantly mediated by perforin and granzyme-A as well granzyme-B [6, 11]. Due to long-term cardiac inflammation, cardiac remodeling and structural changes, CD4^+^CD28^null^ cells are in permanent contact to surface proteins of healthy cardiomyocytes mirroring repeated auto-antigen stimulation. Thus, the CD28^null^ subset gets primed on this specific surface receptor, which now results in auto-antigen reactivity, and consequently cytotoxicity against these structures.

Therefore, the CD28^null^ subset gets primed on this specific surface receptor, now identifying this protein as an “enemy”, tying to eliminate this stimulus via cytotoxicity [6, 12, 13] (see Fig. 1—Sect. [4]):

[6]: As a matter of fact, the release of perforin, granzyme-A and granzyme-B via CD4^+^CD28^null^ cells already showed deleterious effects on smooth vascular muscle cells and cardiac tissue resulting in necrosis and apoptosis [6, 11]. During the initial phase of inflammation T helper cells (CD4^+^) as well cytotoxic T cells (CD8^+^) and NK cells seem to be crucially involved among the inflammatory response.

Of note in patients undergoing cardiac surgery, the fraction of this T cell subset has already been extended during longstanding cardiac disease—the mechanic stimulus of the surgery per se seems to act as a trigger for CD4^+^CD28^null^ cell activation resulting in further myocardial damage. On the other hand—in patients suffering from CHF—continually CD28^null^ cell mediated tissue damage over the course of disease seems be the explanation for the findings of project no.1.

As mentioned above helper cells and as well cytotoxic T cells seem to be crucially involved among the initial phase of inflammation due to IL-2 recruitment. However, their participation on the cardiac inflammation can be limited by IL-10 or TNF-beta mediated maintaining of immunological tolerance and preventing excessive inflammatory response. Therefore, to guarantee a sufficient prevention of excessive inflammatory response the fraction of regulatory T cells seems to expand during the course of disease.

Unfortunately, due to the loss of the CD28 surface protein, the CD4^+^CD28^null^ T cell line is completely resistant to induction of apoptosis and suppression of their immune response by regulatory T cells. This in fact leads to an unimpaired inflammatory state which is further mediated by CD28^null^ cells (4, 14) (see Fig. 1—Sect. [5]):

[6]: Based on deleterious effects of CD4^+^CD28^null^ T cells multiple micro scars among myocardial tissue are formed. Myocardial scars are known to have a major impact on cardiac electric conduction. Therefore, micro scars among cardiac atrial tissue could represent a major trigger for the development of fibrillating impulses and circulating electric cardiac conduction.

Unfortunately, it seems very unlikely, that the inflammatory response is selective to atrial tissue—quite the reverse—a global cardiac inflammation can be assumed. In this context, the different cell-mass comparing cardiac atria to ventricular tissue needs to be considered. Ventricular tissue holds a significantly bigger fraction of the total cardiac mass—which makes it less susceptible for cardiac electric dysfunction based on micro-scars. Therefore, AF seems to mirror the first manifestation of tissue damage due to aggravated CD4^+^CD28^null^ cell mediated cardiac inflammation. However, the fact that patients with CHF are more likely to develop ventricular arrhythmia—such as ventricular tachycardia (VT) or ventricular fibrillation (VF) at the end-staged disease needs to be kept in mind (as a potential ventricular manifestation of aggravated CD4^+^CD28^null^ cell mediated macro-scar formation) (see Fig. 1—Sect. [6]):

[7]: Similarly scars among myocardial tissue are crucially associated with poor contractility of cardiomyocytes. Therefore, the myocardial function decreases, the left-ventricular ejection fraction declines and an increase of myocardial strain can be observed. In this context, we found that the fraction of CD4^+^CD28^null^ cells was directly associated with NT-proBNP—as the most sensitive marker for myocardial strain.

The continuous loss of myocardial function represents the strongest contributor for the overall poor prognosis, resulting in an increased risk for fatal cardiovascular events and the observed increased mortality in individuals with high frequencies of CD4^+^CD28^null^ T lymphocytes. (see Fig. 1—Sect. [7]):

Considering the available data in literature, it needs to be assumed that CD4^+^CD28^null^ T lymphocytes are mainly involved in the development of AF and disease progression. Of utmost importance, it can be considered as the result of a T cell mediated auto-immune reaction among myocardial tissue. However, mechanisms which recruit CD4^+^CD28^null^ cells in cardiac tissue remain unclear and need further investigation. Moreover, further studies addressing the exact pathophysiological mechanism underlying the association of cardiac electric remodeling and CD4^+^CD28^null^ cells are needed to elucidate the potential auto-reactive role of CD4^+^CD28^null^ cells in AF.
